# A Comparison of Psychometric Properties Between Internet and Paper Versions of Two Depression Instruments (BDI-II and MADRS-S) Administered to Clinic Patients

**DOI:** 10.2196/jmir.1392

**Published:** 2010-12-19

**Authors:** Fredrik Holländare, Gerhard Andersson, Ingemar Engström

**Affiliations:** ^4^Swedish Institute for Disability ResearchLinköping UniversityLinköpingSweden; ^3^Department of Behavioural Sciences and LearningLinköping UniversityLinköpingSweden; ^2^Section for Internet PsychiatryDepartment of Clinical NeuroscienceKarolinska InstitutetStockholmSweden; ^1^School of Health and Medical SciencesPsychiatric Research CentreÖrebro UniversityÖrebroSweden

**Keywords:** Questionnaires, psychometrics, Internet, depression

## Abstract

**Background:**

Self-report measures can guide clinical decisions and are useful when evaluating treatment outcomes. However, many clinicians do not use self-report measures systematically in their clinical practice. Internet-based questionnaires could facilitate administration, but the psychometric properties of the online version of an instrument should be explored before implementation. The recommendation from the International Test Commission is to test the psychometric properties of each questionnaire separately.

**Objective:**

Our objective was to compare the psychometric properties of paper-and-pencil versions and Internet versions of two questionnaires measuring depressive symptoms.

**Methods:**

The 87 participating patients were recruited from primary care and psychiatric care within the public health care system in Sweden. Participants completed the Beck Depression Inventory (BDI-II) and the Montgomery-Åsberg Depression Rating Scale—Self-rated (MADRS-S), both on paper and on the Internet. The order was randomized to control for order effects. Symptom severity in the sample ranged from mild to severe depressive symptoms.

**Results:**

Psychometric properties of the two administration formats were mostly equivalent. The internal consistency was similar for the Internet and paper versions, and significant correlations were found between the formats for both MADRS-S (*r* = .84) and the BDI-II (*r* = .89). Differences between paper and Internet total scores were not statistically significant for either questionnaire nor for the MADRS-S question dealing with suicidality (item 9) when analyzed separately. The score on the BDI-II question about suicidality (item 9) was significantly lower when administered via the Internet compared with the paper score, but the difference was small (effect size, Cohen’s [*d*] = 0.14). There were significant main effects for order of administration on both questionnaires and significant interaction effects between format and order. This should not, however, pose a problem in clinical use as long as the administration format is not changed when repeated measurements are made.

**Conclusions:**

The MADRS-S can be transferred to online use without affecting the psychometric properties in a clinically meaningful way. The full BDI-II also seems to retain its properties when transferred; however, the item measuring suicidality in the Internet version needs further investigation since it was associated with a lower score in this study. The use of online questionnaires offers clinicians a more practical way of measuring depressive symptoms and has the potential to save resources.

## Introduction

Routine use of self-report measures (of depressive symptoms, for example) can be useful in clinical settings. A recent study found that clinicians in psychiatric practice rated a self-report measure as helpful for making treatment decisions in 93% of patient visits [1]. In the same study, all the psychiatrists rated the feedback from such rating scales as helpful for monitoring treatment response, and 94% regarded them as helpful for measuring severity of illness. Frequent feedback to clinicians on symptoms from self-report questionnaires has been found to reduce in-patient days and hence the costs of psychiatric care without influencing treatment outcome [2]. According to these results, self-report instruments can be useful tools in mental health care. However, the use of such scales is not the rule in all psychiatric settings. In a survey among UK psychiatrists, only 10.5% reported using self-report scales on a routine basis to measure depression and/or anxiety. Over 55% of psychiatrists answered that they never used self-report scales to measure these symptoms in their patients. One of the most common reasons given for not using self-report was a perceived lack of a robust infrastructure to support the process. In particular, many saw a need for information technology solutions to make such self-report scales more practically useful [3].

One medium that has the potential to make self-report questionnaires easier to use is the Internet. Traditional paper-and-pencil questionnaires are now being complemented by Internet-based questionnaires that can be completed anywhere, reach their destination instantly, automatically calculate scores correctly, and be stored in a practical way. By employing Internet-based questionnaires, clinicians could easily access information about symptom levels and use this information to inform their decisions about treatment. Using the Internet to facilitate this kind of “reflective practice” has been suggested previously [4], and in several treatment studies on Internet-based self-help, patients have used online questionnaires to rate their symptoms [5,6]. Additional possible advantages of Internet-based questionnaires include lower costs, less environmental load, and the ability to rapidly update questionnaires to later versions. More detailed discussions of Internet-based psychological assessments are available [7,8].

There is also an indication that patients may prefer Internet-based questionnaires. One study found that more people reported a preference for responding to mental health questionnaires on a computer compared with answering on paper [9]. Moreover, in a controlled investigation, Internet-based versions of questionnaires yielded higher response rates and fewer missed items compared with the paper versions [10].

Since the spread of the Internet, researchers and test developers have adapted several tests for Internet administration by simply moving the items from established paper questionnaires to websites. However, it has been argued that we cannot assume that the psychometric properties remain the same after such adaptation [11]. The new setting in which the items are presented could render earlier reports on psychometric properties invalid. Internet administration of self-report measures could yield different answers than paper-and-pencil administration. For example, people may tend to answer in a less socially desirable way on the Internet compared with when they answer the same questions on paper [12]. Another reason why Internet administration could differ is computer anxiety, meaning that the use of the computer per se could create some of the very feelings that are rated in questionnaires. One indication of this is a significant correlation between computer anxiety and the score on a scale measuring negative mood that was found when the assessment was made on computer but not when it was done on paper [13]. If scores from Internet versions of self-report measures differ from the established paper versions, it could have serious consequences if decisions about treatment are based on them. This is especially true when it comes to questions about suicidality (item 9 in both the Beck Depression Inventory—second edition (BDI-II) and the Montgomery-Åsberg Depression Rating Scale—Self-rated (MADRS-S) since a score that underestimates the risk might reduce the inclination to ask further questions about suicidal ideation. In Internet-based self-help, possibly underestimating suicide risk becomes even more important since the clinician typically does not meet the patient face-to-face.

The International Test Commission (ITC) guidelines on good practice in Internet-based testing contain recommendations about the process of adapting an established paper-and-pencil questionnaire for online use. The equivalence of the psychometric properties of the two versions is a central issue, and the ITC recommends presenting evidence that the two versions produce scores with comparable means and standard deviations, comparable reliabilities, and a correlation at the expected level from the reliability estimates. It was also stated that there should be the same level of test taker control (for instance the possibility to review or skip items in a similar fashion) [14].

In two previous randomized studies [15,16] that compared Internet and paper versions of the MADRS-S [17], the results indicated similar psychometric properties of the two versions. In spite of these consistent results, there was a need for further investigation mainly because both previous studies used samples with mild depressive symptoms, recruiting only from a university campus in one case [15], and the generalizability of the results is therefore limited. The established paper version of the BDI-II [18] was also compared with Internet versions in the reports of these studies [15,16] as well as with a computerized version in an earlier study with a student sample [19]. There were no differences in the psychometric properties between the two versions in the two studies with samples showing minimal symptoms of depression [15,19], but a significantly higher mean score was generated from the Internet version of the BDI-II compared with its paper version in the study with a sample showing mild depressive symptoms [16]. However, the difference was small.

Previous research on these questionnaires indicates equivalence between Internet and paper versions. Participants in those trials, however, are probably not representative of patients seeking help at psychiatric or primary care clinics, making the results less relevant to this context. From a health care perspective, a study of clinic patients could increase the external validity of a psychometric study, not only because such a study population would consist of persons exhibiting higher levels of depressive symptoms, but also because it is reasonable to assume that clinic patients would have less experience with computers compared with study populations composed of students. Having higher levels of depressive symptoms, individuals in a clinical sample may have some concentration difficulties and therefore might also be more negatively affected by new technology.

The aim of this study was to compare the psychometric properties of two administration formats for the BDI-II and the MADRS-S using a sample of clinic patients. We contrasted paper-and-pencil versus Internet administration of the two questionnaires.

## Method

### Participants

Participants completed the paper and Internet-based questionnaires as part of their registration for a clinical trial of Internet-based treatment for depression. Participants were recruited within public health care in Örebro County Council and Värmland County Council in Sweden. Patients and staff in primary care and psychiatric care were informed about the trial at staff meetings and via posters in waiting rooms. Both referrals and self-referrals were accepted. Participants were required to be at least 18 years of age, have access to a computer with an Internet connection, be fluent in the Swedish language, and be willing to attend two interviews with a psychologist.

### Procedure

All patients who expressed interest in the clinical trial received a letter with an informed consent form. When written consent was received from the patient, the patient was randomized to complete the questionnaires on paper or Internet first to control for order effects. A block randomization sequence was created by a statistician and concealed from study personnel by the use of sealed envelopes. After randomization, a letter was sent to the patient’s home that contained instructions on how to proceed. Patients randomized to completing the paper version first received the questionnaires together with the instructions and a return envelope. Patients randomized to completing the Internet version first received a letter with instructions on how to fill out the Internet version as well as a user name and password. As soon as the trial staff received the responses from the first administration of the questionnaires, a new letter was sent out that contained instructions for the opposite administration format. MADRS-S was completed first and BDI-II second both on the Internet and on paper. Group 1 (n = 43) completed the paper version first and then the Internet version. Group 2 (n = 44) answered the questionnaires in the opposite order. Ethical approval was obtained from the Regional Ethics Committee in Uppsala, Sweden.

### Material

A website was constructed for the study, and all Internet-based measurements were carried out by patients logging in and completing the questionnaires under their user name. All items had to be answered one-by-one, and only one item at a time appeared on the screen. After the answer to an item had been provided, the next item appeared on the screen. It was possible to change the answers of previous items until the last item was completed for each test (BDI-II and MADRS-S).

The Montgomery-Åsberg Depression Rating Scale—Self-rated (MADRS-S) is a 9-item self-report scale that measures depressive symptoms. The patients are asked to rate their symptom severity on a scale ranging from 0 to 6, resulting in a total score ranging from 0 to 54. A higher score indicates a higher level of depressive symptoms. Satisfactory internal consistency was found in a recent study, in which a Cronbach alpha of .84 was reported [20]. The MADRS-S is a self-rated version of the original clinician-rated MADRS, which was especially designed to be sensitive to change in symptom levels [21]. MADRS-S was used in our study because of its briefness, its good psychometric properties, and the fact that it is freely available.

The Beck Depression Inventory—second edition (BDI-II) is a 21-item self-report scale of depressive symptoms. Each item yields a score ranging from 0 to 3 resulting in a total score ranging from 0 to 63, and a higher score indicates a higher level of depressive symptoms. The internal consistency of the BDI-II has been reported to be good in several studies, for example, a Cronbach alpha of .90 has been reported [22]. The BDI-II is a revised version of the original BDI [23]. BDI-II was used in our study because it is one of the most widely used self-report scales for depressive symptoms and is, therefore, of interest to many clinicians.

### Analyses

Cronbach alpha coefficients were used to estimate internal consistency, and Pearson correlations were calculated between the different administration formats. To test differences between the two orders of administration (paper first or Internet first), and formats (paper or Internet) two-way Analyses of Variance (ANOVA) were calculated. Significant interactions were post tested with *t* tests with Bonferroni adjusted alpha levels (*P* < 0.0125). Effect sizes (Cohen’s *d*) were calculated by dividing the difference between scores by the pooled standard deviation.

## Results

Out of 119 patients that showed interest in a clinical trial, 112 gave written consent and were asked to fill out the questionnaires on both Internet and paper. The response rate was 77.7%, that is, 25 patients did not complete the task (4 filled out questionnaires only on the Internet, 8 filled out questionnaires only on paper and 13 didn’t fill out any). A total of 87 patients filled out both questionnaires on paper and on the Internet and are included in the analyses. On average, 9.79 days (SD 9.83) passed between the first and second assessment. Of the 87 study participants, 57 (65.5%) were women, and 30 (34.5%) were men; the mean age was 41.1 years (SD 13.0), ranging from 20 to 72 years of age. The degree of depressive symptoms ranged from minimal to severe, with a range from 7 to 57 on the BDI-II and 6 to 39 on the MADRS-S (paper versions). The mean scores on the paper version of the MADRS-S indicated moderate depressive symptoms, and on the paper BDI-II the mean value indicated severe depressive symptoms.

Cronbach alpha levels were similar for the Internet and paper versions of the Montgomery-Åsberg Depression Rating Scale – Self-rated (MADRS-S). The alpha levels for the different orders and formats of administration are presented in Table 1. The correlation between the MADRS-S total scores from the Internet administration and the paper administration was high *r* = .84 (*P* < .001). Correlations between scores from Internet and paper in the different groups are shown in Table 2. The correlations between the Internet and paper versions of all MADRS-S items were significant. Correlations for each item separately are shown in Table 3.

**Table 1:** Internal consistency (Cronbach alpha) for the two groups and administration formats

	Paper-First Group on Paper Alpha	Internet-First Group on Internet Alpha	Paper-First Group on Internet Alpha	Internet-First Group on Paper Alpha
MADRS-S	.81	.73	.81	.81
BDI-II	.90	.87	.89	.89

**Table 2:** Pearson Correlations between scores from paper and the Internet

	Paper First *r*^a^	Internet First *r*^a^	Both Groups Together *r*^a^
MADRS-S	.86	.80	.84
MADRS-S item 9	.88	.64	.79
BDI-II	.91	.85	.89
BDI-II item 9	.84	.73	.80

^a^All correlations are significant at the *P* < .001 level.

**Table 3:** MADRS-S item, mean score on paper and Internet and the correlation (Pearson) between them

Item	Paper Format Mean (SD)	Internet Format Mean (SD)	Correlation^a^
1 Mood	2.38 (1.73)	2.43 (1.34)	.57
2 Anxiety	3.53 (1.36)	3.55 (1.02)	.58
3 Sleep	2.32 (1.45)	2.32 (1.52)	.66
4 Appetite	1.36 (1.36)	1.48 (1.26)	.65
5 Ability to concentrate	2.83 (1.38)	2.90 (1.32)	.71
6 Initiative	3.18 (1.48)	3.22 (1.43)	.74
7 Emotional involvement	2.66 (1.28)	2.63 (1.21)	.65
8 Pessimism	3.20 (1.38)	3.48 (1.21)	.63
9 Zest for life	2.34 (1.26)	2.41 (1.10)	.79
Total	24.43 (6.97)	23.79 (7.98)	.84

^a^All correlations are significant at the *P* < .01 level.

For the MADRS-S there was no significant main effect for administration format (paper or Internet). There was, however, a significant main effect of administration order, indicating higher scores for the group that answered the questionnaire on paper first compared with the Internet-first group (means 26.2 vs 22.08), and the effect size was moderate (Cohen’s *d* = 0.57). There was also a significant interaction between order of administration and administration format. Subsequent *t* tests with Bonferroni adjusted alpha levels showed no significant difference between scores from paper and Internet for the paper-first group (*t*_42_ = 0.53, *P* = .60), and no significant difference between scores from paper and Internet for the Internet-first group (*t*_43_ = -2.37, *P* = .02). The paper-first group, however, scored significantly higher on the paper-MADRS-S than the Internet-first group (*t*_85_ = 3.1, *P* = .003). No significant difference was found between the Internet scores from the two groups (*t*_85_ = 2.16, *P* = .03).

For MADRS-S item 9 (suicidality) there was no significant main effect for format or order of administration. There was, however, a significant interaction effect between format and order of administration. The subsequent *t* tests showed no significant difference between paper scores and Internet scores in the paper-first group (*t*_42_ = 1.15, *P* = .26), and no significant difference between paper scores and Internet scores in the Internet-first group (*t*_43_ = -1.98, *P* = .05). No significant difference was found between the paper scores from the paper-first and the Internet-first group (*t*_85_ = 2.47, *P* = .02), nor was a significant difference found between the Internet scores from the two groups (*t*_85_ = 1.22, *P* = .23). Means and standard deviations from MADRS-S and BDI-II are shown together with *F* and *P* values for the two groups and administration formats in Table 4.

**Table 4:** Means (SD), main effects, and interaction effect

	Group	Paper Format	Internet Format	Main Effect		Interaction
		Mean (SD)	Mean (SD)	Format F, *P* Value	Order F, *P* Value	F, *P* Value
MADRS-S	Paper first	26.35 (7.93)	26.02 (7.32)			
	Internet first	21.30 (7.28)	22.86 (6.31)	1.88, *P* = .18	7.68, *P* = .007	4.36, *P* = .04
MADRS-S item 9	Paper first	2.67 (1.39)	2.56 (1.24)			
	Internet first	2.02 (1.05)	2.27 (0.92)	0.68, *P* = .41	3.95, *P* = .05	5.1, *P* = .03
BDI-II	Paper first	34.21 (10.9)	31.93 (10.54)			
	Internet first	26.98 (9.34)	27.48 (9.2)	2.97, *P* = .09	7.86, *P* = .006	7.26, *P* = .009
BDI-II item 9	Paper first	0.88 (0.66)	0.72 (0.66)			
	Internet first	0.52 (0.66)	0.5 (0.55)	4.28, *P* = .04	5.08, *P* = .03	2.44, *P* = .12

**Table 5:** BDI-II item, mean score on paper and Internet, and the correlation between them

Item	Paper Format Mean (SD)	Internet Format Mean (SD)	Correlation^a^
(1) Sadness	1.26 (.58)	1.29 (.61)	.70
(2) Pessimism	1.38 (.72)	1.39 (.62)	.66
(3) Feelings of failure	1.55 (.92)	1.53 (.86)	.68
(4) Loss of pleasure	1.72 (.77)	1.59 (.77)	.70
(5) Guilty feelings	1.53 (1.0)	1.53 (.94)	.69
(6) Punishment feelings	0.68 (.97)	0.80 (1.03)	.74
(7) Self-dislike	1.72 (.98)	1.78 (1.02)	.59
(8) Self-criticism	1.43 (.90)	1.43 (.86)	.59
(9) Suicidal thoughts or wishes	0.70 (.68)	0.61 (.62)	.80
(10) Crying	1.68 (1.21)	1.69 (1.19)	.80
(11) Agitation	1.25 (.81)	1.05 (.70)	.66
(12) Loss of interest	1.49 (.83)	1.48 (.85)	.63
(13) Indecisiveness	1.53 (.91)	1.53 (.91)	.71
(14) Worthlessness	1.43 (.90)	1.38 (.90)	.79
(15) Loss of energy	1.84 (.64)	1.68 (.69)	.61
(16) Change in sleeping patterns	1.64 (.85)	1.59 (.87)	.66
(17) Irritability	1.47 (.89)	1.40 (.90)	.68
(18) Changes in appetite	1.39 (.98)	1.22 (.99)	.64
(19) Concentration difficulty	1.52 (.66)	1.44 (.68)	.63
(20) Tiredness or fatigue	1.85 (.77)	1.79 (.88)	.71
(21) Loss of interest in sex	1.48 (1.06)	1.49 (1.04)	.88
Total	30.55 (10.72)	29.68 (10.07)	.89

^a^All correlations are significant at the *P* < .01 level

For the BDI-II Cronbach alpha levels were similar in the Internet and paper versions. The alpha levels for the different orders and formats of administration are presented in Table 1. The correlation between the BDI-II total scores from the Internet administration and the paper administration was high, *r* = .89 (*P* < .001). Correlations between scores from Internet and paper in the different groups are shown in Table 2. The correlations between the Internet and paper versions of all BDI-II items were significant. Correlations for each item are shown in Table 5.

For the Beck Depression Inventory (BDI-II), there was no significant main effect for administration format (paper or Internet). There was, however, a significant main effect for administration order, indicating higher scores for the paper first group compared with the Internet-first group (means 33.07 vs 27.23), and the effect size was moderate (*d* = 0.58). There was also a significant interaction between order and administration format. Subsequent *t* tests with Bonferroni adjusted alpha levels showed that the paper-first group scored significantly higher on the paper BDI than on the Internet BDI (*t*_42_ = 3.36, *P* = .002). No significant difference was found between the paper score and the Internet score for the Internet-first group (*t*_43_ = -0.65, *P* = .52). The paper-first group scored significantly higher than the Internet-first group on the paper BDI (*t*_85_ = 3.33, *P* = .001), but not on the Internet BDI (*t*_85_ = 2.1, *P* = .04).

For BDI item 9 (suicidality), there were significant main effects of format and order of administration, but no significant interaction between them. The mean score for the paper BDI item 9 (both groups) was higher than the Internet BDI item 9 (means 0.7 vs 0.61) and the effect size was small (Cohen’s *d* = 0.14). The paper-first group scored higher (both formats) than the Internet first group (means 0.80 vs 0.51) and the effect size was small (Cohen’s *d* = 0.46).

## Discussion

The internal consistency of both questionnaires was similar across administration formats, and medium to high correlations were found between paper and Internet total scores, and for each individual item. No significant main effect separated the paper total scores from the Internet total scores, but interaction effects were found as well as main effects for order of administration. Participants rated their suicidality on the same level on paper and Internet-based MADRS-S, but rated lower suicidality levels on the Internet BDI-II compared with the paper version.

These results do not indicate any clinically relevant differences between the total scores from paper and Internet versions of the BDI-II and MADRS-S, but rather that people suffering from depression rate their overall depressive symptoms on the same level with both administration formats. An important clinical implication is that it is probable that the questionnaires tested in this study can be used online with the same cutoff points and without changed internal consistency. Online versions should make it easier for clinicians to administer these questionnaires, hopefully making them more common in everyday practice.

If people tend to rate their suicidality lower on the Internet, this has to be taken into account in clinical use. In a previous study [15], however, we did not find a significant difference between suicidality ratings on paper and Internet BDI-II (item 9), and although the difference in the current study was significant, the effect size was small. When it comes to the overall scores, previous research has indicated similar psychometric properties, but in samples with minimal and mild symptoms. Although encouraging, this was of limited clinical relevance since these levels of symptoms are rarely seen in clinical practice. In the current study, the psychometric properties of paper and Internet versions of BDI-II and MADRS-S were compared using a sample of clinic patients recruited within public health care. The sample had mean scores indicating moderate to severe depressive symptoms.

In an earlier study [16] with a sample size of 350, the subjects scored significantly higher on the Internet version than on the paper version of the full BDI-II. In contrast, the current study showed no significant main effect for administration format. The actual difference found by Carlbring et al was small (0.49 points) [16], and thus both studies indicate no clinically meaningful differences between the two administration formats. The difference between the results in the two studies mainly seems to be a difference in statistical power. The significant correlations between scores from Internet and paper versions of each item needs replication since the authors found no previous studies that presented separate results for each item.

A case could be made for a possible difference between the two administration formats, mainly concerning computer anxiety and social disinhibition on the Internet, although this was not directly investigated in the current study. Since no clinically relevant differences were found, these arguments are probably less important in our study. In the case of computer anxiety, a recently published study [24] found that experience with computers reduced the problem, indicating that it is a temporary problem that mainly occurs when new technology is introduced. It is therefore possible that computer anxiety is higher in countries, or subgroups, with low levels of computer experience. In such populations, paper and Internet versions of the same questionnaires may not be equivalent. The design of the current study does not allow any analysis to investigate this.

The significant main effects for order of administration mean that the paper-first group had higher scores regardless of administration format. It is difficult to interpret these results, but one possible explanation is a small difference surrounding the administration of the paper and Internet versions. Before completing the Internet versions, patients had to identify themselves by means of a personal username and password, after which they were asked some questions about sociodemographic characteristics. It is unclear whether this could affect results of both administration formats. Another possible contributing factor could be an actual difference in depressive symptoms between the two groups. The interaction effects found in this study indicate that the order of administration affects the difference between the first and the second measurement if different administration formats are used. In a clinical context it is therefore important to use the same administration format for all measurements made by the same individual.

Since all patients in this study showed an interest in an Internet-based treatment trial, it is possible that they are relatively positive toward using the Internet, which could limit the generalizability of the results. Another limitation of this study is that although the MADRS-S has a maximum score of 54, no subjects in the sample had a score higher than 39 (on the paper version). The full range of the scale was not used and thus the results should not be generalized outside the score range of the sample in the study. A third limitation is that the design did not address the question of test–retest reliability of the Internet versions of the tests. Future studies should address this question by using repeated measures with Internet-based tests. A fourth limitation is that computer anxiety and social disinhibition were not measured. A fifth possible limitation is that the items were presented one at a time on the Internet, which differs from the paper versions. However, earlier research shows that the two methods are psychometrically equivalent [25]. The most apparent strength of the current study is the use of a sample of clinic patients with moderate to severe depressive symptoms.

The results in this study, and in previous studies, suggest that the Internet-based BDI-II generates a total score that does not differ in a clinically meaningful way from the total score generated from the paper version. The suicidality rating in the BDI-II, however, needs further investigation since we found a small but significant difference in this study, but no difference was found in a previous study. Future research on this is needed and should be made with samples with higher levels of suicidality compared with the levels found in this study.

The psychometric properties of MADRS-S were not affected when the scale was transferred for use on the Internet in this study. Since this finding is consistent with two previous studies, it seems safe to transfer the MADRS-S to online use without affecting the psychometric properties in any clinically relevant way. Internet-based MADRS-S is, therefore, a clear candidate to complement traditional self-report measures in clinical work.

Besides the psychometric properties, however, there might also be other problems that have to be addressed before clinical implementation of Internet-based self-report measures, one of which is the security of information technology solutions. Another challenge may be test taker preferences. If patients, or subgroups of patients, find Internet-based questionnaires less attractive than traditional administration formats, it could lower response rates. Future research should investigate the possibilities and challenges associated with implementing online questionnaires in clinical practice. Patient acceptability, information security, and cost effectiveness are some important aspects.

## Abbreviations

BDI-II: Beck Depression Inventory—Second Edition
ITC: International Test Commission
MADRS-S: Montgomery-Åsberg Depression Rating Scale—Self-rated
